# Gold Nanoparticles for Photothermal Cancer Therapy

**DOI:** 10.3389/fchem.2019.00167

**Published:** 2019-04-05

**Authors:** Jeremy B. Vines, Jee-Hyun Yoon, Na-Eun Ryu, Dong-Jin Lim, Hansoo Park

**Affiliations:** ^1^Casinbio USA, Birmingham, AL, United States; ^2^Department of Herbology, College of Korean Medicine, Woosuk University Jeonju, South Korea; ^3^School of Integrative Engineering, Chung-Ang University Seoul, South Korea; ^4^Otolaryngology Head and Neck Surgery, University of Alabama at Birmingham Birmingham, AL, United States

**Keywords:** gold, photo-active property, hyperthermia, nanoparticles, cancer therapeutics

## Abstract

Gold is a multifunctional material that has been utilized in medicinal applications for centuries because it has been recognized for its bacteriostatic, anticorrosive, and antioxidative properties. Modern medicine makes routine, conventional use of gold and has even developed more advanced applications by taking advantage of its ability to be manufactured at the nanoscale and functionalized because of the presence of thiol and amine groups, allowing for the conjugation of various functional groups such as targeted antibodies or drug products. It has been shown that colloidal gold exhibits localized plasmon surface resonance (LPSR), meaning that gold nanoparticles can absorb light at specific wavelengths, resulting in photoacoustic and photothermal properties, making them potentially useful for hyperthermic cancer treatments and medical imaging applications. Modifying gold nanoparticle shape and size can change their LPSR photochemical activities, thereby also altering their photothermal and photoacoustic properties, allowing for the utilization of different wavelengths of light, such as light in the near-infrared spectrum. By manufacturing gold in a nanoscale format, it is possible to passively distribute the material through the body, where it can localize in tumors (which are characterized by leaky blood vessels) and be safely excreted through the urinary system. In this paper, we give a quick review of the structure, applications, recent advancements, and potential future directions for the utilization of gold nanoparticles in cancer therapeutics.

## Introduction

### Current Limitations in Conventional Cancer Therapies

In 2017, cancer was the second-most common cause of death in the United States, comprising 22.5% of the total number of deaths; 591,699 people died from complications related to cancer in 2017 (Heron, 2018). Unfortunately, owing to the heterogeneous nature of cancer, there are currently no fully comprehensive approaches for treatment; options are mainly limited to chemotherapy, radiotherapy, immunotherapy, and surgery. Although these approaches provide some therapeutic efficacy, they are limited by their risk to normal, healthy cells, their potential to destroy the immune system, or by conferring an increased risk for the development of secondary cancers (Nolsoe et al., 1993; Vogel and Venugopalan, 2003; Kievit and Zhang, 2011). For this reason, a large body of cancer therapy research focuses on finding effective therapies that can complement or even replace current therapies by improving efficacy and reducing inadvertent side effects.

### Hyperthermia as a Cancer Treatment Modality

In the pursuit of therapies capable of reducing undesired side effects and enhancing efficacy, there has been growing interest in utilizing hyperthermia to achieve these goals. Hyperthermic cancer therapy was originally developed based on historical examples in which cancer patients infected by erysipelas had high fevers that either reduced cancer symptoms or resulted in the complete regression of tumors (Moyer and Delman, 2008). Since the original study pioneered by Coley in 1893, additional studies have been performed in which hyperthermia was carefully applied to general regions of cancerous tumor growth to maintain tissue temperatures of around 42 to 45°C (Luk et al., 1980). In practical settings, however, the ability to precisely manage heating surrounding the tumor area would be critical to improve and adapt this alternative modality on cancer treatment. Additionally, other cancer treatment modalities such as radiation and chemotherapy can be utilized together for successful cancer treatment. In this regard, not only does hyperthermia cause apoptosis of cancer cells, but can also improve therapeutic efficacy when used in concert with radiation or chemotherapy (Kampinga, 2006). In the presence of thermal stress, tumors become radiosensitized, making them more likely to respond to radiotherapy, resulting in improved cancer survival rates. This fact has been demonstrated in studies of metastatic head and neck squamous cell cancers, where radical radiation treatment with hyperthermia resulted in improved outcomes without increasing toxicity (Moyer and Delman, 2008; Kaur et al., 2011). A similar sensitization is further seen with chemotherapeutics when used in combination with hyperthermia. When clinically-relevant drugs for malignant melanoma are combined with low or high grade hyperthermia (43 and 45°C, respectively), intrinsic or extrinsic ER-mediated apoptosis can be induced (Mantso et al., 2018).

Many preclinical studies have been performed to demonstrate that both radio and chemotherapy can be enhanced by simultaneously incorporating hyperthermic therapy (Peeken et al., 2017). Unfortunately, traditional hyperthermia techniques are not ideal due to the fact that they are not minimally invasive, and result in the non-specific generation of heat throughout the body (Kaur et al., 2016). As a result, substantial undesirable side effects are created. For example, whole-body hyperthermia may cause cardiovascular side effects and gastrointestinal symptoms (Chatterjee et al., 2011). Regarding this, a more promising modality for cancer treatment would involve a targeted, nanoparticle-mediated localized hyperthermia One treatment modality that continues to gain attention and is currently under investigation for potential widespread use is photothermal therapy (PTT) (Bardhan et al., 2011; Melancon et al., 2011). Photothermal therapy is based on the conversion of light energy (usually in the near-infrared region) into heat energy to induce subsequent cellular necrosis or apoptosis (Ray et al., 2012).

Compared with other methods, light is an ideal external stimulus as it is easily regulated, focused, and remotely controlled. This ease of focus and control enable better targeted treatments that lead to less damage to healthy tissues (Yang X. et al., 2012; Khaletskaya et al., 2013; Zhu et al., 2014). Unfortunately, traditional photodynamic therapy (PDT) of tissues mediated by laser or visible light is limited by insufficient depth of penetration, limiting its usefulness for deep tumor therapy (Ochsner, 1997; Wilson and Patterson, 2008; Benov, 2015). However, near-infrared (NIR) light (in the wavelength range of 800–1,200 nm) has much greater body transparency, making it preferable for PTT. In contrast to traditional PDT, which relies on the presence of oxygen to generate reactive oxygen species, and is considerably limited in application due to its limited depth of penetration (Wilson and Patterson, 2008), PTT mainly exerts effects by increasing the local temperature within tumors (Wang and Qiu, 2016). Regarding this, it has been demonstrated that in order to completely destroy cancer cells *in vitro*, a threshold temperature ranging between 70 and 80°C is required (Huang et al., 2006). Furthermore, at temperatures ranging from 55 to 95°C, tumorigenic damage is evident *in vivo* conditions (Thomsen, 1991).

### Hyperthermic Nanoparticle Systems and Limitations

Some current nanoparticle technologies for hyperthermic therapy include ferromagnetic nanoparticles such as iron oxide (van Landeghem et al., 2009; Wang et al., 2010; Cassim et al., 2011; Maier-Hauff et al., 2011), doped iron oxide (Lee et al., 2011; Fantechi et al., 2014; Gordon et al., 2014) and super-paramagnetic iron oxide nanoparticles (SPION) (Le Renard et al., 2010; Kruse et al., 2014; Zheng et al., 2014) as well as carbon nanotube (CNT) technologies including single walled carbon nanotubes (SWCNTs) and multi-walled carbon nanotubes (MWCNTs) (Burke et al., 2009; Huang et al., 2010) in addition to various polymer-based technologies (Kaur et al., 2016).

Ferromagnetic nanoparticles including SPION, iron oxide, and doped iron oxide are typically stimulated under the presence of alternating magnetic fields (AMFs) during which materials are induced to rapidly magnetize and demagnetize. When these materials are manufactured as nanoparticles, their magnetization rapidly fluctuates generating a net field of zero (superparamagnetism). When superparamagnetic nanoparticles are stimulated with magnetic fields they behave like paramagnets with a single magnetic domain and enhanced magnetic susceptibility. Upon the application of an AMF, superparamagnetic nanoparticles can be reasonably excited to generate heat sufficient for thermal therapy. The main limitation of the magnetic nanoparticle approach is the fact that it is difficult to generate fine-tuned and precise treatment of tumors due to the fact that AMF fields are generally targeted toward the whole body in contrast to the tumor specifically as seen with photothermal approaches (Dennis et al., 2008).

CNTs are nanomaterials that are composed of sheets of carbon atoms arranged into the shape of a honeycomb-like lattice that are rolled into the shape of a tube only a few nanometers in diameter but with lengths anywhere on the scale of hundreds of nanometers to microns (Kaur et al., 2016). SWCNTs are made up of one CNT while MWCNTs are comprised of multiple tubes stacked within each other. CNTs are capable of responding to light across a broad-spectrum including light in both the visible and NIR spectrums. Previous studies have shown the successful utilization of SWCNTs for the treatment of squamous cell carcinoma tumor xenografts in mice using NIR illumination (Huang et al., 2010) and the successful utilization of MWCNTs in concert with short pulses of low-power laser illumination for the treatment of renal cancer xenografts (Burke et al., 2009). However, one of the main limitations associated with CNTs is the fact that granulomas resembling asbestos associated mesothelioma in the mesothelial and pleural linings have occasionally presented in mice, raising concerns regarding their long-term biocompatibility (Poland et al., 2008).

There are also currently many polymeric materials geared toward applications in PTT. To date, polypyrrole, poly-(3,4-ethylenedioxythiophene):poly(4-styrenesulfonate) (PEDOT:PSS), dopamine-melanin (polydopamine), and polyaniline nanoparticles are some of the most commonly used materials that have been reported to show photothermal effects (Chen et al., 2012; Cheng et al., 2012; Yang K. et al., 2012; Liu et al., 2013; Vines et al., 2018).

Perhaps one of the oldest conducting polymers employed for PTT is known as Polyaniline (Zhou et al., 2013). Its low cost, mechanical flexibility, and excellent conductivity has provided this material with considerable recognition (Li et al., 2009). In addition, polyaniline has historical use as an electroactive tissue for studying cellular proliferation prior to its utilization in PTT due to its excellent biocompatibility (Heeger, 2001). Another of the most commonly used base materials for use in PTT cancer treatments is Polypyrrole (PPy) (Wang, 2016; Manivasagan et al., 2017). PPy, which was originally known as “pyrrole black” due to its composition as a black precipitate from acidic pyrrole/ H_2_O_2_ aqueous solutions was first synthesized in the early 20th century. PPy has recently found popularity as an electro-responsive material in biomedical engineering applications (Ateh et al., 2006; Svirskis et al., 2010; Balint et al., 2014) as it is generally regarded as biocompatible, with little or no adverse effects on health (George et al., 2005; Fahlgren et al., 2015).

Poly(3,4-ethylenedioxythiophene):poly(4-styrenesulfonate) (PEDOT:PSS) is another class of polymer-based nanoparticles commonly used for NIR-mediated hyperthermic therapy. In perhaps the first documented study of this material for photothermal cancer therapy, PEGylated PEDOT:PSS nanoparticles (PDOT:PSS-PEG) were synthesized via a layer-by-layer approach, creating nanoparticles of ~80 nm in diameter (Cheng et al., 2012). Polydopamine, another commonly utilized polymer for PTT, was first explored as a potential PTT agent by Liu et al. (2013). In this study, dopamine-melanin colloidal nanospheres were fabricated via the oxidation and self-polymerization of dopamine in a mixture containing water, ethanol, and ammonia at room temperature.

While these polymer based nanoparticle systems show some promise, many polymer based systems such as polydopamine hold less than ideal mass extinction coefficients (Dong et al., 2016). In addition to sometimes holding weaker photothermal efficiencies, the degradation profiles of many of these polymers is not fully understood, creating questions as to their long-term biocompatibility (Cheng et al., 2014). For this reason, it may be beneficial to utilize nanomaterials with longer documented historical utilization in clinical practice.

### Gold in Medicine and Hyperthermic Cancer Therapeutics

Gold (Au), one of the noble metals, has been characterized by its resistance to corrosion and oxidation. These properties have been known for centuries, as evidenced by gold's long-documented use in medicinal applications. Colloidal Au was documented in the Middle Ages as a substance for treatment and diagnosis of diseases (Pricker, 1996). Inspired by the early discovery of the bacteriostatic properties of K[Au(CN)_2_] (Shaw, 1999), gold compounds were eventually utilized for modern medical treatments. Recent advancements in nanomedicine have recognized the use of Au in the therapeutic delivery of drugs or as a therapeutic modality in itself. For example, colloidal Au is covalently linked onto adenoviral vectors for selective cancer targeting and induces hyperthermia by application of near-infrared (NIR) laser light (Everts et al., 2006).

Recent advancements in the multi-functional design of gold nanoparticles allow for the generation of localized heat in the proximity of cancer tissues and additionally allow the delivery of multiple desired drugs in a controlled and targeted manner. Gold nanoparticles have many benefits that make them suitable for the photothermal treatment of cancer such as: (1) they can be administered into the local tumor area while minimizing non-specific distribution, (2) they can be activated via near-infrared (NIR) laser light, creating the ability to penetrate deep into biological tissues, and (3) they can be modulated to create multifaceted cancer PTT and drug delivery systems (Kennedy et al., 2011).

### Gold's Localized Surface Plasmon Resonance (LSPR) as a Distinctive Photo-Active Property

**Figure 1:**
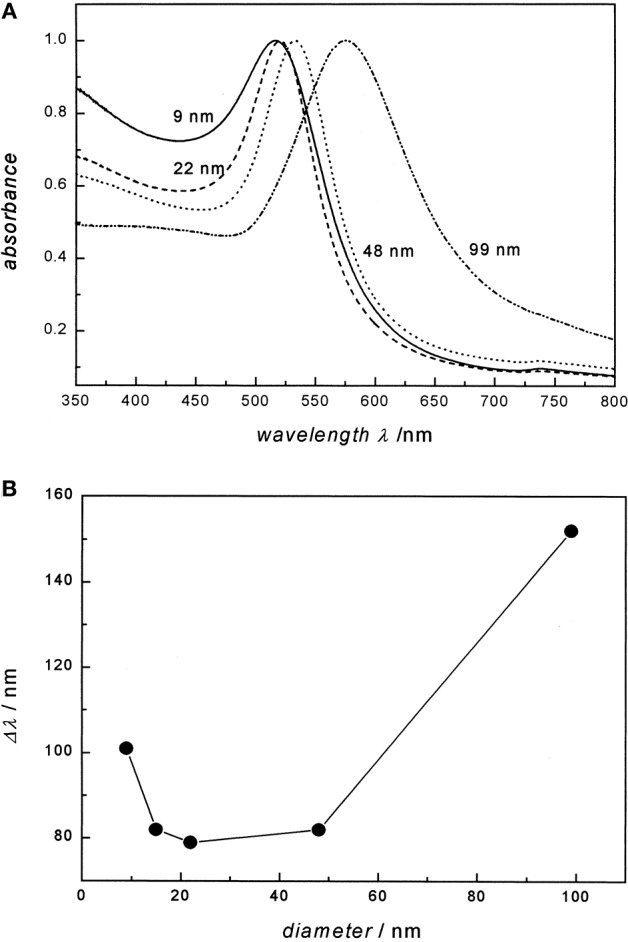
Particle diameter of Au on the absorption spectra and the plasmon bandwidth. **(A)** UV/ Visual absorption spectra of 9, 22, 48, and 99 nm gold nanoparticles in water. All spectra are normalized at their absorption maxima, which are 517, 521, 533, and 575 nm, respectively. **(B)** The plasmon bandwidth Δλ as a function of particle diameter. Re-printed with permission from American Chemistry Society Publications 1999 (Link and El-Sayed, 1999).

Colloidal Au exhibits a unique localized surface plasmon resonance (LSPR) when a specific wavelength of light meets electrons on the surface of gold. LSPR is defined as an optical phenomenon where interactions occur between the incident light and surface electrons in a conduction band (Petryayeva and Krull, 2011). The light causes a collective coherent oscillation of conduction band electrons, leading to the subsequent extinction of light. Scattering and absorption of the light depends not only on the physical dimensions of the gold nanoparticle but also on the medium of the colloidal Au (El-Sayed, 2001; Kelly et al., 2003). Small colloidal Au absorbs the blue-green portion of the visible spectrum and visible light in the red portion of the visible light spectrum. However, in large colloidal Au, the LSPR results in the absorption of longer wavelengths of light along the red portion of the VLS, resulting in the reflection of light in the blue spectrum. A study of the LSPR spectra of different colloidal Au also indicates a shift toward the red spectrum. For example, 22 nm of colloidal Au in water exhibited a maximum absorption spectrum at 517 nm. However, when 99 mm of colloidal Au is present, there is a significant shift toward the red end of the spectrum (Link and El-Sayed, 1999). It is therefore known that the plasmon bandwidth of the gold nanoparticles is affected by the particle diameters of colloidal Au (Figure 1).

**Figure 2:**
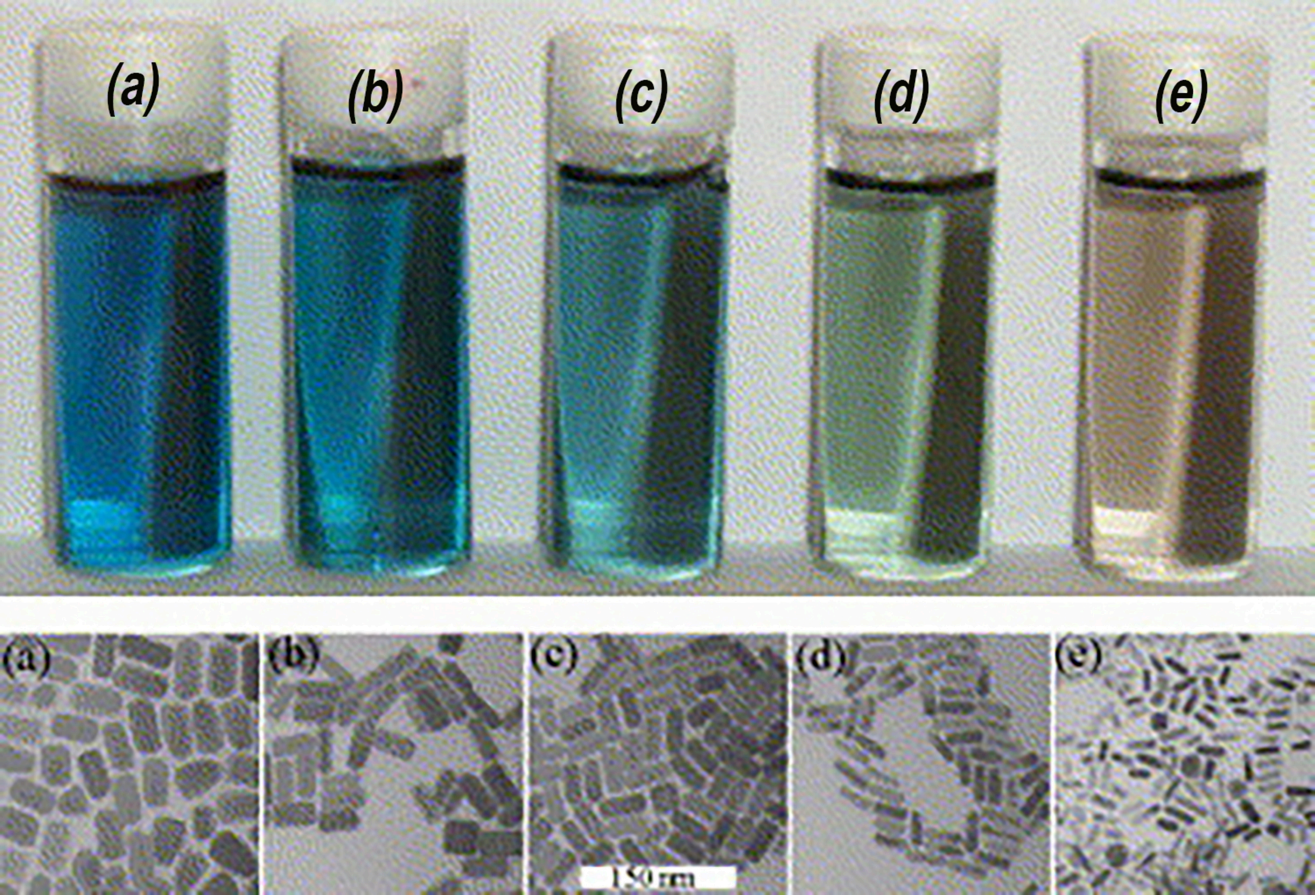
Color of gold nanorods with different aspect ratios. The small difference in the aspect ratio shows distinctive transmitted colors in the samples. Re-printed with permission from Elsevier 2005 (Pérez-Juste et al., 2005).

**Figure 3:**
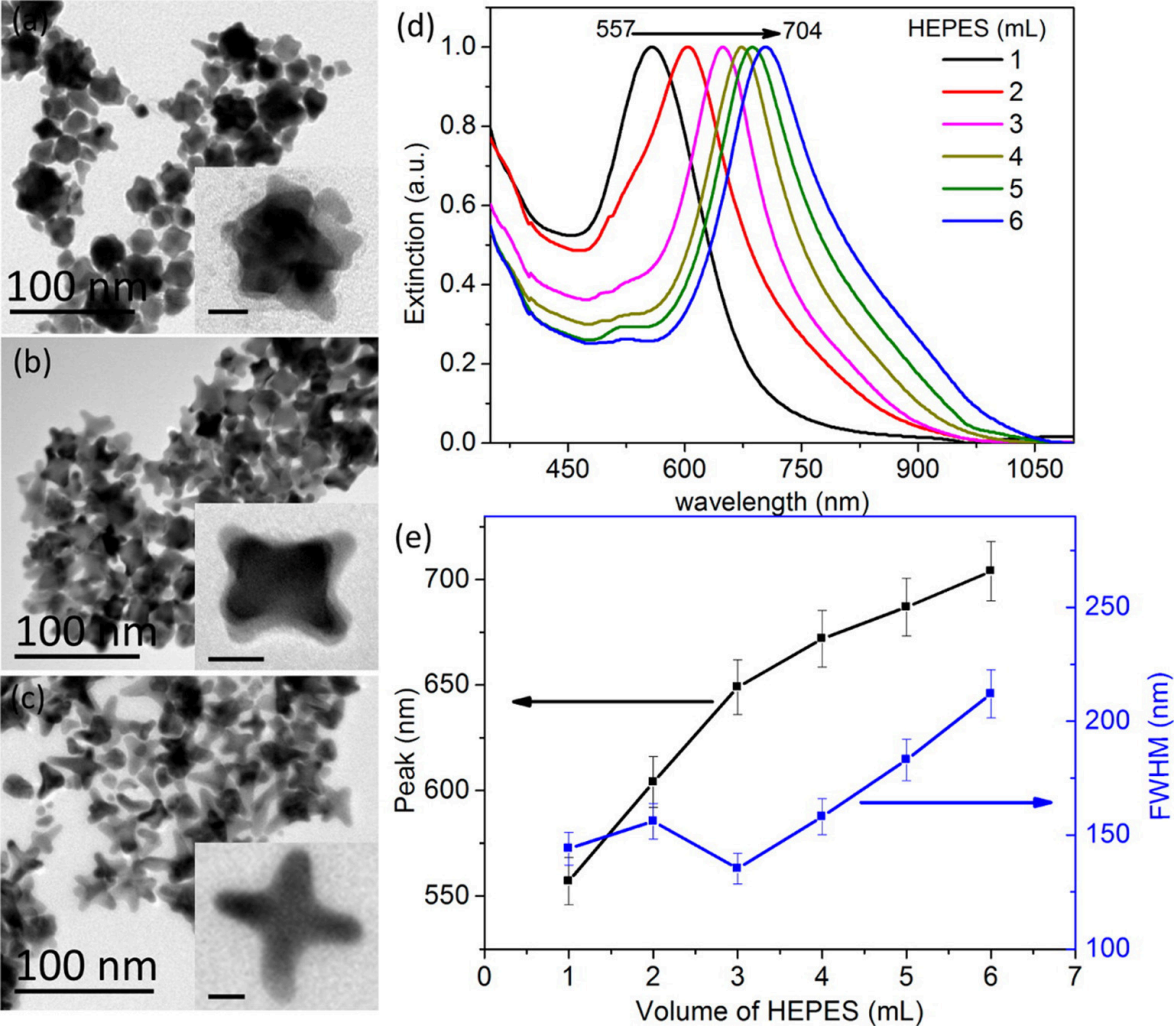
Fabrication of gold nanostars with different shapes. TEM images of gold nanostars prepared at 30°C are shown in the left panel. The authors were able to create different shapes by adding **(a)** 1, **(b)** 3, and **(c)** 6 mL of HEPES solution (0.1 M). The scale bar indicates 10 nm. **(d)** Normalized extinction spectra of gold nanostars with different volumes of HEPES. **(e)** The main extinction peak and FWHM (full width at half-maximum) as a function of HEPES volume. Re-printed with permission from American Chemistry Society Publications 2014 (Liu et al., 2014).

To modulate the LSPR of the gold, several different shapes and sizes of gold have been studied. Gold (Au) nanorods (GNRs) are able to present longitudinal and transverse surface plasmon absorption peaks (Smitha et al., 2013). The length of Au nanorods presents the longitudinal resonance whereas the transverse resonance is attributed to the diameter of the GNRs. It is well-known that the spectral location of the LSPR can be modulated by changing the aspect ratio of GNRs (Smitha et al., 2013). GNRs with different aspect ratios (length/width) create different-color nanorod solutions due to changes in their reaction with light in the visible light spectrum (Figure 2) (Pérez-Juste et al., 2005). Similarly, three-tipped Au nanoparticles fabricated using a wet technique showed a significant red-shift compared to spherical-shaped gold nanoparticles (Hao et al., 2004). The branched colloidal Au particles exhibited a plasma band between 650 and 700 nm, while the maximum absorption spectrum for regular, spherical shaped colloidal Au was between 500 and 530 nm. Because the resonant excitation of plasmons is affected by the surface of nanoparticles, a gold nanostar as defined by a solid core with protruding prolate tips, can exhibit hybridized plasmons because they are made with a solid core that has tips (Hao et al., 2007). Liu et al. manipulated the growth of gold nanostars using a 4-(2-hydroxyethyl)-1-piperazineethanesulfonic acid (HEPES) solution, which acts as a reducing and capping agent for gold nanocrystals (Figure 3) (Liu et al., 2014). The fabricated gold nanostars demonstrated a red-shift from 557 to 704 nm. The more HEPES solution was added, the more reducing power was exhibited in the growth of gold branches, appearing at up to 20 nm in length. The elongated Au branches resulted in enhanced longitudinal plasmon resonances.

**Figure 4:**
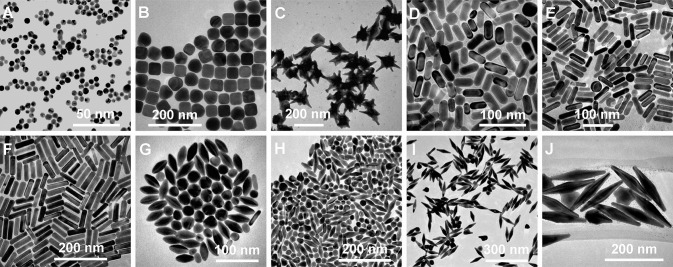
Diversity of gold nanostructures. **(A)** Nanospheres. **(B)** Nanocubes. **(C)** Nanobranches. **(D)** Nanorods (aspect ratio = 2.4 ± 0.3). **(E)** Nanorods (aspect ratio = 3.4 ± 0.5). **(F)** Nanorods (aspect ratio = 4.6 ± 0.8). **(G)** Nanobipyramids (aspect ratio = 1.5 ± 0.3). **(H)** Nanobipyramids (aspect ratio = 2.7 ± 0.2). **(I)** Nanobipyramids (aspect ratio = 3.9 ± 0.2). **(J)** Nanobipyramids (aspect ratio = 4.7 ± 0.2). Re-printed with permission from American Chemistry Society Publications 2008 (Chen et al., 2008).

**Figure 5:**
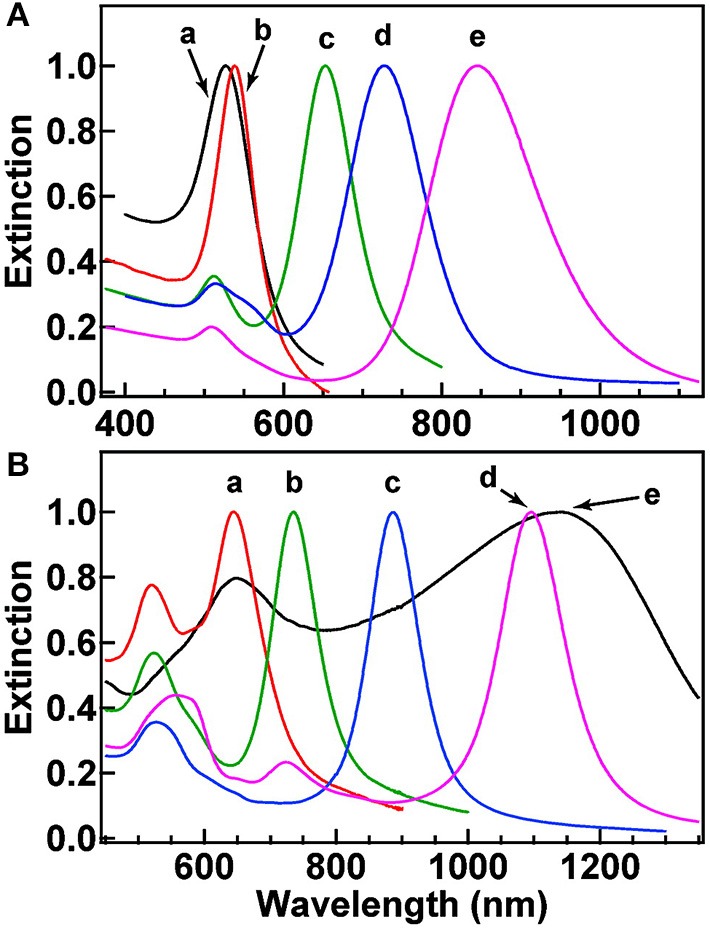
Normalized extinction spectra of the gold nanostructures. **(A)** Spectra a–e correspond to nanospheres (aspect ratio = 2.4 ± 0.3), nanocubes (aspect ratio = 3.4 ± 0.5), and nanorods with aspect ratios of 4.6 ± 0.8. **(B)** Spectra a–d correspond to nanobipyramids with different aspect ratios (as shown 1.5 ± 0.3, 2.7 ± 0.2, 3.9 ± 0.2, and 4.7 ± 0.2, respectively) and nanobranches (spectra e). Re-printed with permission from American Chemistry Society Publications 2008 (Chen et al., 2008).

Another unique shape of Au capable of tuning LSPR that has been developed is Au nanorings. Au nanorings with diameters between 75 and 150 nm were fabricated using colloidal lithography (Larsson et al., 2007). The LSPR of different nanorings between 75 and 150 nm in diameter was between 1,000 and 1,300 nm, indicating that the diameter of the ring-like Au structures contributed to the tunability of the Au nanostructures. Using different fabrication methods, nanospheres, nanocubes, nanobranches, nanorods, and nanobipyramids were prepared and characterized for LSPR (Figure 4) (Chen et al., 2008). As expected, Au nanospheres and nanocubes exhibited one surface plasmon peak, whereas nanobranches, nanorods, and nanobipyramids exhibit two major surface plasmon peaks. There is a consistent LSPR shift toward light in the red spectrum from nanospheres (Figure 5A wave a), nanocubes (Figure 5A wave b), and nanorods with different aspect ratios (Figure 5A wave c–e). The larger aspect ratio of the nanorods exhibits a longer red-spectrum shift in the nanorods (Figure 5A wave c–e, the aspect ratios were 2.4 ± 0.3, 3.4 ± 0.5, and 4.6 ± 0.8, respectively). Exhibiting a similar pattern, nanobipyramids with different aspect ratios also show a red spectrum shift (Figure 5B wave a–d). The highest red spectrum shift was found in fabricated nanobranches due to their considerable longitudinal electron oscillation (Figure 5B wave e).

## Gold Nanoparticle Synthesis

Gold nanoparticles are synthesized via either physical or chemical approaches wherein either a bottom-up or top-down approach is taken (Cunningham and and Bürgi, 2013; Aminabad et al., 2018). Bottom-up methods typically involve the nucleation of gold on top of smaller structures using either chemical, electro-chemical, or thermal reduction techniques (Singh et al., 2011; Cunningham and and Bürgi, 2013; Shah et al., 2014).

The most commonly used of the bottom-up techniques is the Turkevich and Brust method, wherein metal salts are reduced in order to produce spherical, monodisperse GNPs around 10–20 nm in diameter (Cunningham and and Bürgi, 2013; Shah et al., 2014). Sodium citrate salts are commonly used to serve as both a reducing agent and stabilizer that acts to prevent GNP aggregation during synthesis (Zare et al., 2010). In lieu of citrate, ascorbic acid, amino acids, and UV light have all been used as reducing agents (Mieszawska et al., 2013). Schiffrin-Brust is an early, two-phase procedure employing tetrabutylammonium bromide (TOAB) to transfer gold from organic to inorganic solutions, enabling the synthesis of GNPs in organic solutions with high stability (Li et al., 2011; Herizchi et al., 2016). Using this method, GNPs ranging from 2 to 6 nm in diameter can be synthesized.

The most commonly employed Top-down techniques usually create nanoscale materials through the processing of larger macroscale structures via techniques such as lithography (Cunningham and and Bürgi, 2013). Other commonly employed physical synthesis methods include sonochemical, microwave, and photochemical based methods (Herizchi et al., 2016). A recently developed technique utilizes N-cholyl-L-valine (NaValC) as a self-reducing and stabilizing agent intended to be coupled with natural sunlight irradiation for the synthesis of GNPs (Annadhasan et al., 2015). By modifying the ratio of Au^3+^ to NaValC ions, the amount of sunlight irradiation, pH, and the reaction time, the size and shape of synthesized GNPs can be changed.

Recently, a new fabrication method was developed in which aqueous [AuCl_4_] can be irradiated with 532 nm nanosecond laser pulses to produce monodisperse 5 nm GNPs without the utilization of capping agents or additives, eliminating the possibility of contamination by residual chemicals (Rodrigues et al., 2018). Five hundred and thirty two nanometer nanosecond laser irradiation results in a more uniform monodispersion of 5 nm diameter GNPs compared to older methods using 800 nm femtosecond laser irradiation, which generally results in the growth of nanoparticles as large as 40 nm.

## Gold Nanoparticles for Efficient Cancer Therapy

Gold nanoparticles (GNPs) have been investigated in the context of various cancer therapies and are sought after as a potential alternative or adjunct to many non-selective chemotherapeutic agents as a means by which to improve therapeutic outcomes while reducing undesirable side effects (Jain et al., 2012). The efficacy of plasmonic gold nanoparticles for the thermo-ablation of various cell types has been demonstrated in multiple studies. The efficacy of gold nanoparticles for the thermal-mediated induction of cellular death was demonstrated by Pitsillides et al. (2003), wherein anti-CD8-labeled GNPs were used for the selective targeting and destruction of T-cells (Pitsillides et al., 2003).

### Biocompatibility of Gold Nanoparticles

GNPs are considered non-cytotoxic overall with the expectation that despite their small size (2–4 nm), they are likely to be rapidly excreted via the kidneys (Longmire et al., 2008; Alric et al., 2013). In terms of localized non-specific cytotoxicity, study results are mixed, with some studies demonstrating no cellular toxicity and others demonstrating the production of cellular reactive oxygen species, apoptosis, necrosis, and acute mitochondrial toxicity (Shukla et al., 2005; Pan et al., 2009; Balasubramanian et al., 2010). Sufficient accumulation of GNPs inside the body can cause nontoxicity, as demonstrated by the fact that when GNPs accumulate within the liver, tissue apoptosis, acute inflammation, and an increase in Kupffer cells can occur (Longmire et al., 2008; Chen et al., 2009; Cho et al., 2009; Khlebtsov and Dykman, 2011). However, this effect is largely size-dependent, as smaller GNPs (<8 nm) are capable of passing through the renal filtration system whereas larger GNPs (>10 nm) are more likely to remain in the blood stream, and thus accumulate in the liver and kidney (Bartneck et al., 2012; Zhang et al., 2012; Blanco et al., 2015). It is therefore known that the toxicity of GNPs greatly depends on their specific size and configuration.

The effect of GNPs on the immune system likely depends on their configuration, with one study showing that GNPs can induce pro-inflammatory responses contingent on their size (Yen et al., 2009) and other studies showing anti-inflammatory responses (Tsai et al., 2012; Sumbayev et al., 2013). In these studies, a consistent theme is the role of GNP size on the nature and scale of the inflammatory response with one study illustrating that nanoparticles of 5 nm in size were capable of significantly inhibiting production of IL-1B in THP-1 derived macrophages with 35 nm sized nanoparticles demonstrating no effect (Sumbayev et al., 2013). A similar study showed that 4 nm diameter GNPs inhibited inflammatory responses in RAW269 derived murine macrophages via the inhibition of TLR9 responses, likely by binding and interfering with high-mobility group box-1 (Tsai et al., 2012). In contrast, the enhanced inflammatory response exhibited in another study can be explained by the fact that the sizes were larger on average, with sizes ranging from 14 to 100 nm, with larger sizes demonstrating the greatest upregulations in IL-1, IL-6, and TNF-alpha (Yen et al., 2009).

### Surface Modification of Gold Nanoparticles for Specific Tumor Targeting

Because of the leaky nature of immature vasculature found at the sites of tumors, GNPs can passively accumulate at tumor sites, where they are likely taken into cells via non-specific receptor-mediated endocytosis (RME) (Maeda, 2001; Chithrani et al., 2006). However, while GNPs may be capable of passive delivery to tumor sites to some extent, there are still limitations owing to the heterogeneity of vasculature in different types of cancers. Passive delivery is also further inhibited by particles and uptake on behalf of the reticuloendothelial system (RES) (Fang et al., 2011). Therefore, more specific methods for the targeted delivery of GNPs to sites of tumor growth are necessitated.

GNPs also exhibit unique physiochemical properties such as the ability to bind thiol and amine groups along with surface plasmon resonance (SPR), which allows their specific modification for more targeted cancer therapies (Shukla et al., 2005). This property enables the implementation of surface modifications that can enhance passive cellular uptake. One such method is known as PEGylation, which can be achieved by using thiol-terminated methoxypoly (ethylene glycol) to replace the stabilizing surfactant bilayers that normally surround GNPs (Liao and Hafner, 2005). By modifying the surface of GNPs with polyethylene glycol, cellular uptake may be enhanced due to the affinity of PEG for cellular membranes (Choi et al., 2003; Paciotti et al., 2006).

In one study demonstrating this principle, pH-sensitive, multifunctional gold nanocomposites were created by conjugating the anti-cancer drug doxorubicin hydrochloride to GNPs using Adamantane-PEG(8)-RGDS molecules, thus creating AuNP@CD-AD-DOX/RGD GNPs. The RGD peptide sequence was included to target the alphavbeta3 integrin, which is known to be overexpressed on the surface of cancer cells, therein facilitating receptor-mediated endocytosis of the GNPs into the cancer cells. Following cellular uptake and internalization into endo/lysosomes, the hydrazine linkage between adamantane and doxorubicin is cleaved owing to acid-mediated degradation. Experiments demonstrated the uptake of AuNP@CD-AD-DOX/RGD gold nanoparticles and subsequent release of DOX once internalized into cellular endo/lysosomes, resulting in the induction of apoptosis within cancer cells (Chen et al., 2015).

For example, more specific targeting of tumors can be achieved by conjugating tumor-specific recognition molecules such as transferrin, folic acid, epidermal growth factor (EGF), or any number of monoclonal antibodies to the surface of GNPs (El-Sayed et al., 2005; Chithrani et al., 2006; Eghtedari et al., 2009). This strategy has been employed with promising effects in multiple studies. In one study, citrate-coated GNPs were conjugated with trastuzumab (anti-EGF receptor monoclonal antibodies) to target EGF receptors in human SK-BR-3 breast cancer cells, resulting in downstream expression of EGF receptors and a 2-fold increase in trastuzumab cytotoxicity, even at low GNP concentrations (Jiang et al., 2008). In another study, GNPs were conjugated to gemcitabine and cetuximab for the treatment of pancreatic cancer (Patra et al., 2010). Furthermore, phase II trials using this combination have been in clinical trials for this purpose (Kullmann et al., 2009). By using this targeted approach, it was shown that it is possible to utilize much higher concentrations of GNPs while simultaneously avoiding substantial accumulation of nanoparticles within the liver and kidneys (Patra et al., 2008).

In a recent study by Kim et al. NIR plasmonic gold nanoparticles possessing both photothermal and photoacoustic properties allowing for both enhanced contrast imaging and therapeutic applications were developed (Kim et al., 2017). To accomplish this, bioconjugates were created in which human methyl binding domain protein 1 (MBD1) binds to methylated cytosine-guanine dinucleotides (mCGs) within sequences of short double-strand DNA (sh-dsDNA), with hexahistidine peptides serving as nucleation sites for GNP synthesis. The synthesis of these hybrid GNP constructs called DMAs (sh-dsDNA-MBD1-AuNPs), allowed for the modification of photothermal and photoacoustic properties by changing the length of the sh-dsDNA backbone. Three sh-dsDNA backbone lengths were investigated (DMA_5mCG, DMA_9mCG, and DMA_21mCG). Interestingly, the DMA_21mCG conjugate exhibited similar photothermal properties and, surprisingly, higher photoacoustic properties compared to regular plasmonic gold nanorods. By further conjugating peptide sequences with a specific affinity to EGF receptor, it is possible to target cancer cells overexpressing the EGF receptor.

Considering that an important component of tumor progression is the ability of many cancers to evade and suppress the host's immune system (Kim et al., 2006; Finn, 2008), finding methods to improve the ability of the immune system to target cancers is of growing interest. Various factors such as nanoparticle shape, charge, particle size, and coating can influence their blood clearance and organ accumulation, with smaller particles and coated particles exhibiting an ability to more widely distribute within the body (Sonavane et al., 2008; Almeida et al., 2011, 2014; Hirn et al., 2011; Khlebtsov and Dykman, 2011). GNPs are further known to accumulate in organs such as the liver and spleen, where they are likely to interact with the patient's immune system (Zhang et al., 2009). Considering that GNPs are known to accumulate within immune cells, the utilization of GNPs as a drug delivery methodology for immunotherapy has seen an increase in interest.

Because of their strong SPR, GNPs are consistently considered for use in photodynamic therapy (PDT), where light-induced heating can be exploited to either induce heating to release a chemical payload or to generate reactive oxygen species to induce either cellular necrosis or apoptosis at specific tumor sites (Harris et al., 2006; Pissuwan et al., 2006, 2009; Norman et al., 2008). In one study, a 4-component antibody-phtalocyanine-polyethyline glycol-gold nanoparticle conjugate was developed for use in a PDT approach to target breast cancer. Zinc-phtalocyanine, a known photosensitizer, was conjugated to GNPs along with Anti-HER2 monoclonal antibodies, which are known to target cancer cells overexpressing the HER2 epidermal growth factor cell surface receptor (Stuchinskaya et al., 2011). Experiments demonstrated the ability of the nanoparticle conjugates to selectively target and induce the apoptosis of breast cancer cells.

## Commonly Used Gold Nanoparticle Configurations

While tissues have limitations related to PDT mediated by ether laser light or light within the visible spectrum due to limited depth of penetration (Ochsner, 1997; Wilson and Patterson, 2008; Benov, 2015), near-infrared light in the range of 800–1,200 nm has much greater body transparency. Fortunately, by modifying the shape of GNPs, such as by using GNRs or hollow gold nanoshells, their resonance peak can be shifted toward the NIR spectrum (Loo et al., 2004). In this regard, various gold nanoparticle configurations have been employed to modify their photothermal and subsequently therapeutic efficiencies (Vats et al., 2017).

### Gold Nanospheres

Gold nanospheres (GNS) are perhaps one of the earliest GNP shape configurations to be studied, with some of the first demonstrations of the use of GNS for PTT being performed by El-Sayed et al. (Huang et al., 2008). GNS were popularized by their ease of fabrication, small size, fast synthesis, and ease of ligand conjugation, making them attractive for PTT applications (Day et al., 2010). Various modified forms of gold nanospheres have been shown to exhibit therapeutic properties when conjugated to antibodies targeting tumors overexpressing specific proteins (Day et al., 2010), and have been modified with other metals to improve their photoacoustic and photothermal properties (Zhang et al., 2015). Thermo-labile liposome-based GNS (LiposAu NPs) have also been developed for the purpose of cancer photo thermal therapy. The bioabsorbable core of these liposome-based gold nanospheres provides a beneficial structure that allows for more efficient body clearance of the gold via hepato-biliary and renal routes (Rengan et al., 2014, 2015).

### Gold Nanostars

Gold nanostars have recently gained notoriety because of their enhanced NIR light-absorbing capability in addition to their reduced toxicity (Chen et al., 2015). Further, their thin, branch-like structure gives them tip-enhanced plasmonic properties (Ahmad et al., 2016). Many studies have demonstrated the successful utilization of multifunctional gold nanostars for photothermal applications using NIR wavelength light in the targeting of various types of cancer cells in various modified forms (Chen et al., 2013; Gao et al., 2015; Li et al., 2016). In one study, octahedral solid core Au nanohexapods were fabricated by reducing HAuCl4 with DMF in an aqueous solution containing Au octahedral seeds (Wang et al., 2013). Relative to gold nanorods (GNRs) and nanocages, PEGylated nanohexapods demonstrated the greatest tumor uptake and photothermal conversion efficiency.

### Gold Nanoshells

Gold nanoshells are another widely utilized GNP configuration. The gold nanoshell structure consists of dielectric silica gels that are encased within a thin, hollow, outer gold shell (O'Neal et al., 2004). By modifying shell thickness and core diameter, it is possible to configure gold nanoshells to absorb light in the NIR spectrum, making them suitable for photothermal and photoacoustic applications (Hirsch et al., 2003). Various surface modifications have been applied to gold nanoshells to functionalize them for anti-tumor therapy. To encourage natural accumulation of nanoparticles at tumor sites, West et al. synthesized PEGylated gold nanoshells by conjugating nanoshells with PEG-SH (O'Neal et al., 2004). For example, anti-EGFR antibodies have been conjugated to a nanoshell platform for breast cancer therapy (Loo et al., 2005). In another study, branched nanostructure gold nanoshells with PLGA/DOXO-cores underwent a tri-modal modification consisting of functionalization with human serum albumin/indocyanine green/folic acid applied to enhance both their targeting and enhanced photothermal properties on account of the NIR-responsive indocyanine green dye (Topete et al., 2014).

Gold nanoshell configurations provide unique flexibility due to their method of fabrication, allowing them to mimic the specific aspect ratios of other nanomaterial configurations such as nanorods to enhance properties such as cellular uptake and increasing drug loading capabilities due to higher superficial surface areas. As a recent study demonstrates, rod-like, gold nanoshell mesoporous silica nanoparticles (MSNR@Au hybrid) were fabricated and further functionalized via modification with ultrasmall gadolinium (Gd) chelated supramolecular photosensitizers TPPS4 [MSNR@Au-TPPS4(Gd)] to enable quadmodal imaging with near-infrared fluorescence (NIRF), multispectral optoacoustic tomography (MSOT), computed tomography (CT), and magnetic resonance (MR) in addition to its inherent NIR driven photothermal capability (Yang et al., 2019).

### Gold Nanorods

Gold nanorods (GNRs) were first synthesized by Wang et al. (Chang et al., 1997), with the first documented use of nanorods for use in NIR spectrum photothermic therapy being reported in 2006 by El-Sayed et al. (Jain et al., 2006). The unique shape of GNRs confers strong photothermal properties due to the presence of both longitudinal and transverse plasmon (Hwang et al., 2014). These strong photothermal properties have been used for anti-tumorigenic applications, many times utilizing various surface modifications such as conjugating surface antibodies for specific targeting, utilizing dendrimer stabilization, or even developing a chitosan oligosaccharide surface modification (Charan et al., 2012; Wang et al., 2016). In one study performed by Cui et al. GNRs were loaded onto induced pluripotent stem cells (AuNR-iPS) for the purpose of targeting human gastric cancer cells. It was demonstrated that AuNR-iPS were able to localize to human gastric cancer tumors and induce thermal-mediated apoptosis and reduction in tumor volume following NIR irradiation (Liu et al., 2016).

GNRs appear to be among the most utilized GNP configurations, with consistent development of GNR based photothermal technologies demonstrated in recent history. In one such recent study, inorganic phototherapeutic nanocomplexes were created by conjugating GNRs with defective TiO2 nanoparticle clusters (AuNR-TiO2 NP clusters) to reduce the need for organic photosensitizers, which are sensitive to photobleaching and unnecessary energy transfer (Lee et al., 2018). These nanoparticle clusters were capable of absorbing both visible and NIR light from the 500 to 1,000 nm spectrum, demonstrating the ability to induce cell death in HeLa cells via the photothermal production of ROS. Notable derivatives of GNR configurations are also coming to the forefront of photothermal cancer therapeutics. In another recent study, the lumens of halloysite nanotubes (HNTs) were loaded with GNRs and doxorubicin (DOX) following which, the GNR filled HNTs were conjugated with folic acid using bovine serum albumin for more specific tumor targeting (Au-HNTs-DOX@BSA-FA) (Zhang et al., 2019). By combining the chemotherapeutic approach of DOX with the photothermal capability of GNRs, it was possible to reduce collateral damage to healthy tissues by DOX while still achieving the same therapeutic outcomes.

### Other Gold Nanoparticle Configurations

The development of new GNP configurations continues to evolve as new synthesis methodologies are continually developed. As an example of a recently developed technology, Zhang et al. exploited extracellular vesicles to generate popcorn-like gold nanostructures (Zhang et al., 2019). These extracellular vesicles enabled the encapsulation of DOX while serving as a nucleation site for gold nanoparticle shells, allowing for simultaneous photothermal transduction and chemotherapeutic potential. Overall, this technology provided a novel modality for the green synthesis of GNPs while improving cellular internalization allowing for tumor inhibitory rates of up to 98.6%.

Gold nanoflowers (GNFs) are a unique GNP configuration that was recently developed by Li et al. (2015). This technology takes advantage of the superior photothermal conversion efficiency of gold nanostars over GNRs and gold nanoshells while also providing a hollow core structure to enhance therapeutic efficiency by enabling chemotherapeutic drug loading. Some recent modifications of the GNF technology include the encapsulation of ultrasmall iron oxide nanoparticles for multimodal image therapy (Lu et al., 2018) and self-assembly using vapreotide acetate (Vap) for enhanced photothermal conversion efficiency as well as enhanced biocompatibility (Yin et al., 2018).

## Future Directions: Green Synthesis of Gold Nanoparticles Incorporated With Natural Substances

Isolation and fabrication of gold nanoparticles from natural substances may provide some benefits over traditional synthesis methodologies. It is speculated that the green synthesis of gold nanoparticles using natural substances may enhance their medical properties such as their anti-microbial and anti-cancer activity, and contribute to reducing and stabilizing agents for the synthesis of nanoparticles (Kumar and Yadav, 2008). Synthesis of GNPs in this fashion is considered more cost-effective and may result in the production of GNPs that have fewer to no side effects due to the reduction of residual chemicals necessary for gold nanoparticle synthesis. The most prominent sources for the green synthesis of GNPs include Bacterium, Fungi, and Plants (Table 1).

**Table 1:** Green chemistry for gold nanoparticle synthesis.

	**Source**	**Size (nm)**	**Morphology**	**References**
Bacteria	*Bacillus megatherium* D01	1.9 ± 0.8	Spherical	Iravani, 2014
	*Bacillus subtilis* 168	5–25	Octahedral	
	*Escherichia coli* DH5α	25 ± 8	Spherical, triangular, and quasi-hexagonal	
	*Escherichia coli* MC4100	10–25	Spherical, triangular, hexagonal, and rod shape	
	*Geobacillus* sp.	5–50	Quasi-hexagonal	
	*Lactobacillus* strains	20–50	Crystalline, hexagonal, triangular, and cluster	
	*Plectonema boryanum* UTEX 485	10 up to 6 μm	Cubic and octahedral platelet	
	*Pseudomonas fluorescens*	50–70	Spherical	
	*Rhodopseudomonas capsulata*	10–20	Nanoplate and spherical	
Fungi	*Fusarium oxysporum*	8–40	Spherical	Mukherjee et al., 2002
	*Verticillium* sp.	5–200 (average 20 ± 8 nm)	Spherical	Mukherjee et al., 2001
Plant	Apiin extracted from henna leaves	7.5–65	Quasi-spherical	Iravani, 2011
	*Camellia sinensis* (green tea)	40	Spherical, triangular, irregular	
	*Coridandrum sativum* (coriander)	6.75–57.91	Spherical, triangular, truncated triangular, decahedral	
	*Cymbopogon flexuosus* (lemongrass)	200–500	Spherical, triangular	
	*Eucalyptus camaldulensis* (river red gum)	1.25–17.5	Crystalline, spherical	
	*Medicago sativa* (alfalfa)	2–40	Irregular, tetrahedral, hexagonal platelet, decahedral, icosahedral	
	*Mentha piperita* (peppermint)	150	Spherical	
	*Murraya koenigii*	20	Spherical, triangular	
	*Ocimum sanctum* (tulsi; leaf extract)	30	Crystalline, hexagonal, triangular	
	Pear fruit extract	200–500	Triangular, hexagonal	
	*Pelargonium graveolens* (geranium)	20–40	Decahedral, icosahedral	
	*Psidium guajava* (guava)	25–30	Mostly spherical	
	*Scutellaria barbata* D. Don (Barbated skullcup)	5–30	Spherical,triangular	
	*Sesbania drummondii* (leguminous shrub)	6–20	Spherical	
	*Syzgium aromaticum* (clove)	5–100	Crystalline, irregular, spherical, elliptical	
	*Tamarindus indica* (tamarind)	20–40	Triangular	
	*Terminalia catappa* (almond)	10–35	Spherical	
	*Trichoderma koningii*	30–40	Triangular	

Extracts from the leaves of *Catharanthus roseus* (CR) and *Carica papaya* (CP), which contain active components associated with the treatment and prevention of cancer, were conjugated to gold nanoparticles. It is likely that the stabilizing molecules were alkaloids, flavones, and proteins that are present on the leaf extracts. The biogenic gold nanoparticles have demonstrated a consistent ability to negatively influence the viability of HepG2 liver cancer cells and MCF7 breast cancer cells due to the synergism of delivery with gold nanoparticles and the anti-cancer activity of the plant extracts. The anti-bacterial activity of the gold nanoparticles was also investigated against gram positive bacteria. However, it was noted that gold nanoparticles are considered to have greater activity against gram negative bacteria, meaning that the results may have underestimated their anti-bacterial properties (Muthukumar et al., 2016).

In another study, gold nanoparticles were fabricated and conjugated with baicalin, which is an active flavonoid that can be found in *Scutellaria baicalensis* and has anti-cancer properties. The gold particles synthesized by baicalin demonstrated cytotoxicity against the MCF7 cell line. Western blot analysis showed greater expression of Aparf-1 and cleaved capase-3 bands in the cell groups treated with baicalin-complexed gold nanoparticles compared to controls, indicating that the baicalin-conjugated gold nanoparticles negatively influenced breast cancer growth by inducing apoptosis (Lee et al., 2016). In another study, croin, which is the main carotenoid found in Saffron stigma (*Crocus sativus*), which exhibits antioxidative activity, mediated the reduction reaction of Au 3^+^ ions performing fast formation gold nanoparticles of controlled sizes. Gold nanoparticles conjugated with croin effectively suppressed the proliferation of breast cancer cells in a time- and dose-dependent manner. The study also demonstrated that there was no cytotoxic effect against normal cells (MCF-10A) (Hoshyar et al., 2016).

Gold nanoparticles conjugated with plant extracts derived from the leaves and stems of *Hibiscus sabdariffa* were reported to have selective cytotoxic activity against U87 glioblastoma (GNB) cells. The cellular viability of normal 293 cells and U87 GMB cells treated with gold nanoparticles was analyzed using an MTT assay. The MTT assay results demonstrated that there was a dose-dependent cytotoxicity against U87 GMB cells; however, there was no significant toxicity detected among normal cell lines. Further, it was demonstrated that a concentration of 2.0 ng/mL of biogenic gold nanoparticles induced cell death of more than 80% of cancer cells under both normal and hyperglycemic conditions. In addition, cells treated with a concentration of 2.5 ng/mL of gold nanoparticles demonstrated degradation of GAPDH (glyceraldehyde-3-phosphate dehydrogenase), which is known to be over-expressed in cancer (Mishra et al., 2016).

Overall, the utilization of natural derivatives as an adjunct to cancer therapy using gold nanoparticles appears to be a promising approach for the selective targeting of tumors while subsequently reducing any side effects that may be incurred via the utilization of synthetic drug compounds.

## Limitations of Gold Nanoparticles for PTT

To be considered an ideal candidate for PTT, a specific set of requirements should be met. For example, the ideal PTT candidate should be: (i) of a suitable nanoparticulate size and of uniform shape; (ii) have good dispersibility in aqueous solutions; (iii) respond to light in the NIR range of 650–950 nm to prevent damage to surrounding healthy tissues, provide sufficient photothermal efficiency, and to enable sufficient depth of penetration; (iv) be sufficiently photostable to ensure adequate diffusion time to reach tumors before losing their photosensitivity, (v) exhibit low or no cytotoxicity in living systems (Zhou et al., 2013).

While GNPs fulfill most of these requirements, its long-term cytotoxicity is largely unknown. As discussed earlier, while GNPs are considered to be largely biocompatible, the long-term consequences of nanoparticle accumulation are not fully understood. However, there are some initial studies hinting at potential factors that can influence GNP cytotoxicity. Based on these studies, it is believed that size and surface charge are likely to be the most influential factors. For example, it was demonstrated that 46% of the initial dose of 5 nm positively charged gold-dendrimer complex particles were excreted after 5 days. In contrast, another study found that for 5 nm particles that were negatively or neutrally charged or for nanoparticles measuring above 11 nm, only about 10% of the initial dose was excreted (Balogh et al., 2007). The areas with the largest accumulation tend to be the liver and spleen, with one study finding foreign bodies in 7 out of 8 spleens and 8 out of 8 livers from animals that received intravenous injection of PEG-coated GNRs (Goodrich et al., 2010). It was speculated that these foreign bodies arose from the aggregation of GNRs in these tissues. Furthermore, evidence of chronic inflammation characterized as minimal to mild was observed in the areas around these foreign bodies, although the long-term consequences of this inflammation were not clear from the study.

Unfortunately, studies on GNPs have only taken place in animal models up to a 6-month time-frame, leaving unanswered questions as to how GNPs influence health over longer time courses. Therefore, while initial studies are promising regarding issues of potential cytotoxicity, there are still questions as to whether GNPs eventually clear from the body and if there are potentially long-term consequences resulting from GNP accumulation (Goodrich et al., 2010).

In addition to the fact that the issue of biocompatibility surrounding GNPs is not completely resolved, there are other currently existing technologies that could potentially make the utilization of GNPs obsolete. As an example, the utilization of specific biodegradable polymer systems for PTT has grown in prominence. A recent study highlights the use of a novel polymer based photothermal nanoagent capable of responding to light in the NIR-II spectrum (1,000–1,700 nm) (Sun et al., 2018), which is capable of much greater depth of tissue penetration than light in the NIR-I spectrum. However, it should be noted that GNPs could conceivably be modified via conjugation with NIR-II responsive polymers, although the potential advantages of doing so over using pure NIR-II polymer nanoparticles has not yet been explored.

## Conclusion

Because of its bacteriostatic, anti-oxidative, and anti-corrosive properties, gold has been utilized for medical applications dating back centuries. In addition, its photothermal and photoacoustic properties, along with its ability to be manufactured at the nano-scale and functionalized with various drugs and targeting molecules, have caused gold nanoparticles to be recognized as an ideal multifunctional material for cancer therapeutics. Because of its successful documented use in *in vitro*, pre-clinical, and clinical studies, it has been demonstrated that GNP technology is a promising tool and it is worth investigating future directions that would allow for a further evolution of the use of GNPs for cancer therapeutics.

## Author Contributions

JV performed literature search as well as the majority of the authoring and editing. J-HY performed the literature search and writing for Future Directions. N-ER, D-JL, and HP proposed topic of paper and provided overall direction of manuscript. D-JL proposed figures and helped author introduction sections.

### Conflict of Interest Statement

The authors declare that the research was conducted in the absence of any commercial or financial relationships that could be construed as a potential conflict of interest.
